# Alcoholic hepatitis and metabolic disturbance in female mice: a more tractable model than *Nrf2*^−/−^ animals

**DOI:** 10.1242/dmm.046383

**Published:** 2020-12-29

**Authors:** Lozan Sheriff, Reenam S. Khan, Raquel Saborano, Richard Wilkin, Nguyet-Thin Luu, Ulrich L. Gunther, Stefan G. Hubscher, Philip N. Newsome, Patricia F. Lalor

**Affiliations:** 1 Centre for Liver and Gastroenterology Research, Institute of Immunology and Immunotherapy, University of Birmingham, Birmingham B15 2TT, UK; 2 Birmingham National Institute for Health Research (NIHR) Birmingham Biomedical Research Centre, Institute of Immunology and Immunotherapy, University of Birmingham, Birmingham B15 2TT, UK; 3 Institute of Cancer and Genomic Sciences, University of Birmingham, Birmingham B15 2TT, UK; 4 Institute of Chemistry and Metabolomics, University of Lübeck, 23562 Lübeck, Germany; 5 Liver Unit, University Hospitals Birmingham, Birmingham B15 2TH, UK; 6 Department of Cellular Pathology, University Hospitals Birmingham, Birmingham B15 2TH, UK

**Keywords:** Alcoholic hepatitis, Inflammation, Fibrogenesis, Steatosis, Murine

## Abstract

Alcoholic hepatitis (AH) is the dramatic acute presentation of alcoholic liver disease, with a 15% mortality rate within 28 days in severe cases. Research into AH has been hampered by the lack of effective and reproducible murine models that can be operated under different regulatory frameworks internationally. The liquid Lieber-deCarli (LdC) diet has been used as a means of *ad libitum* delivery of alcohol but without any additional insult, and is associated with relatively mild liver injury. The transcription factor nuclear factor-erythroid 2-related factor 2 (*Nrf2*) protects against oxidative stress, and mice deficient in this molecule are suggested to be more sensitive to alcohol-induced injury. We have established a novel model of AH in mice and compared the nature of liver injury in C57/BL6 wild-type (WT) versus *Nrf2*^−/−^ mice. Our data showed that both WT and *Nrf2*^−/−^ mice demonstrate robust weight loss, and an increase in serum transaminase, steatosis and hepatic inflammation when exposed to diet and ethanol. This is accompanied by an increase in peripheral blood and hepatic myeloid cell populations, fibrogenic response and compensatory hepatocyte regeneration. We also noted characteristic disturbances in hepatic carbohydrate and lipid metabolism. Importantly, use of *Nrf2*^−/−^ mice did not increase hepatic injury responses in our hands, and female WT mice exhibited a more-reproducible response. Thus, we have demonstrated that this simple murine model of AH can be used to induce an injury that recreates many of the key human features of AH – without the need for challenging surgical procedures to administer ethanol. This will be valuable for understanding of the pathogenesis of AH, for testing new therapeutic treatments or devising metabolic approaches to manage patients whilst in medical care.

This article has an associated First Person interview with the joint first authors of the paper.

## Abstract (precis)

**Summary:** We describe a simple murine model of alcoholic hepatitis (AH) to induce injury that recreates many of the key features of AH in human, without the need of challenging surgical procedures to administer ethanol. This will be valuable for testing new therapeutic treatments.

## INTRODUCTION

Alcoholic liver disease (ALD) places an enormous burden on patients, their carers and society as a whole. In the past 30 years, mortality from ALD has risen by 450% (https://acmedsci.ac.uk/file-download/34760-CallingT.pdf), and in part this relates to the more dramatic, acute form of disease, alcoholic hepatitis (AH). There is no approved therapy for AH and, although drugs, including immune modulators and monoclonal antibodies, have been tested for management of severe AH, only glucocorticoids and pentoxyfylline are currently licensed for use (European Association for the Study of the Liver, 2012). However, two meta-analyses of pentoxyfylline use did not show convincing benefits (Louvet et al., 2018; Thursz et al., 2015). Similarly, although the large randomised, controlled STeroids Or Pentoxifylline for Alcoholic Hepatitis (STOPAH) trial showed that corticosteroids can improve short-term survival (28 days) in patients with severe AH, 40% of patients were considered ‘non-responders’ (Mathurin, 2005). Furthermore, no significant mortality benefit was seen following the application of steroids at 90 days or one year in the STOPAH trial (Thursz et al., 2015). Importantly, steroids have significant side effects, including infection (Hmoud et al., 2016), sepsis, haemorrhage and hepato-renal syndrome. Therefore, the UK NIHR James Lind Alliance has identified AH as a priority translational research area owing to the lack of licensed therapies.

Research into new therapies to treat AH has been disadvantaged by the lack of effective murine models that recreate the features of the human disease, whilst being reproducible and acceptable to local regulatory policies. The pathogenesis of AH is multifactorial, and characterised by macrovesicular steatosis, infiltration of immune cells and hepatocellular damage accompanied by an increase in serum transaminases. The inflammatory response is associated with impaired hepatocyte regeneration in response to injury. Additional histological changes include perivenular and pericellular fibrosis. Although a number of rodent models have been used to study AH, all have their individual challenges and deficiencies. Chronic *ad libitum* ethanol (EtOH) feeding using a Lieber–DeCarli (LdC) liquid diet has been widely tested but generates a relatively mild injury without fibrosis when used alone (Han et al., 2013; Hu et al., 2013; Kirpich et al., 2012; Li et al., 2014; Zhang and Meadows, 2008). A number of two-hit models attempted to generate more-severe injury by combining administration of the LdC diet with, e.g. the use of genetically modified mice (Ji et al., 2006, 2004; Ki et al., 2010; Ronis et al., 2015), alcohol gavage (Chang et al., 2015; Lazaro et al., 2015) and lipopolysaccharide (LPS) (Kong et al., 2017; Wieser et al., 2017). The National Institute on Alcohol Abuse and Alcoholism (NIAAA) developed a model of AH that involves chronic EtOH feeding during an LdC diet, followed by a single large binge of EtOH by gavage (Bertola et al., 2013b). This model does induce a robust neutrophil-mediated liver injury but the high-dose ethanol binge causes mice to become moribund, which is internationally inacceptable within all regulatory systems. Similarly, models that involve continuous intragastric EtOH infusion (Tsukamoto et al., 1985a) are associated with severe hepatic steatohepatitis and mild fibrosis; but, complex surgery, single-mouse housing and intensive monitoring are beyond remit for many facilities, and not amenable to regulatory drug screening.

Thus simpler, less-challenging models that recreate hepatic and systemic features of human AH are required for widespread use. One option is to incorporate enhanced hepatic oxidative stress to generate a more-significant injury than that generated by ethanol administration alone. In this context, the nuclear factor-erythroid 2-related factor 2 (Nrf2)-deficient mouse has been investigated. Nrf2 is a transcription factor considered the ‘master regulator’ of anti-oxidant defences. Lamlé and colleagues suggested that *Nrf2*-knockout mice (*Nrf2^−/−^*) developed a more-severe hepatic injury than wild-type (WT) mice given high-dose gavage (Lamlé et al., 2008). Thus, we have adapted this model and describe a reproducible protocol that causes robust steatosis, elevation in serum transaminase levels and hepatic inflammation. However, by using this dietary protocol, we also show that, in our hands, the use of *Nrf2^−^^/−^* mice offers no advantage compared with female WT animals. Importantly, we report systemic responses (weight loss and neutrophilia) and hepatic metabolic changes (elevated taurine, lipid intermediates and lactate) (Brandl et al., 2018; Ding et al., 2019; Latchoumycandane et al., 2014) as well as characteristic histological features (steatosis and neutrophil infiltration) that provide insight into the steatogenic mechanisms that characterise AH. Thus, in conclusion, we suggest that administration of a modified LdC diet to female WT mice represents a tractable and simple model of AH when combined with daily ethanol gavage (hereafter referred to as LdC+EtOH diet).

## RESULTS

### Exposure of WT and *Nrf2^−/−^* mice to LdC+EtOH causes inflammation, and hepatic steatosis with early fibrogenic change

**Fig. 1.**
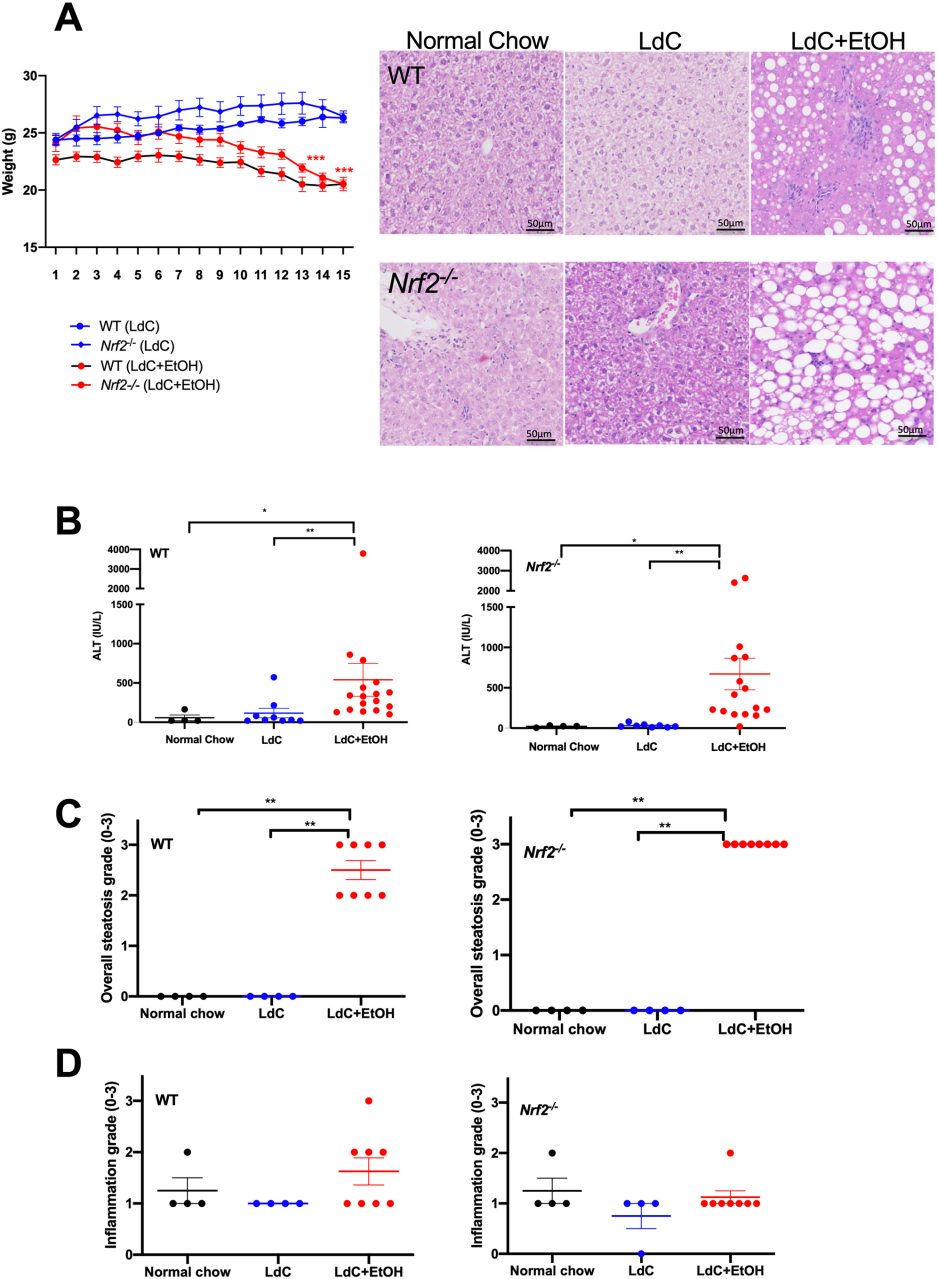
**Mice fed an LdC diet** **and ethanol exhibit weight loss, inflammation and hepatic steatosis.** WT and *Nrf2^−/−^* mice were fed LdC diet only for 5 days. At day 6 experimental groups received 2.2% EtOH mixed into the LdC diet. This was increased to 4.4% at day 9, and to 6.3% at day 12. Additionally, experimental groups received twice daily ethanol gavage of 33% EtOH (LdC+EtOH). Control mice received LdC diet only throughout the experiment and were not gavaged. (A) The graph indicates mean mouse weight ±s.e.m. (in g). Linear regression analysis confirmed a significant weight loss in WT and *Nrf2*^−/−^ mice on LdC+EtOH (*P*<0.01 for both). Representative H&E-stained liver sections from WT and *Nrf2^−/−^* mice that had been fed normal chow, LdC or LdC+EtOH are shown on the right. Scale bars: 50 μm, data are representative of at least *n*=6 animals per group. (B) Serum was prepared from WT and *Nrf2^−/−^* mice at day 15 to determine the concentration of ALT. Circles represent values from individual mice as indicated, bars indicate the mean±s.e.m. for the group. Statistical differences are *P<0.05 and **P<0.01, obtained using the Kruskal–Wallis Dunn's multiple comparison test. (C) H&E-stained sections were scored semi-quantitatively for the grade of hepatic steatosis on a scale of 0-3. Circles represent values from individual mice and bars indicate the mean±s.e.m. for each group. Statistical differences are *P<0.05 and **P<0.01, obtained using the Kruskal–Wallis Dunn's multiple comparison test. (D) H&E-stained sections were scored for total inflammation on a grade of 1-3. Circles represent values from individual mice and bars indicate the mean±s.e.m. of the group.

To induce a reproducible alcohol injury in mice, we modified an LdC diet (Preedy et al., 1988; Wilkin et al., 2016) feeding protocol. Female WT and *Nrf2^−/−^* mice were fed liquid LdC diet alone for 5 days to acclimatize prior to the addition of escalating doses of ethanol into the diet (up to a final concentration of 6.2% at day 12). An additional twice daily gavage of 33% of EtOH diluted with H_2_O was added from day 6 until day 15. Control mice were fed an LdC diet only throughout the experiment. Although the mean weight of the experimental and control groups was similar at baseline, both WT and *Nrf2^−/−^* mice lost a significant amount of weight after introduction of EtOH, with weight loss approaching 20% by day 15 (Fig. 1A). At day 15, both WT and *Nrf2^−/−^* mice exposed to LdC+EtOH showed significantly increased levels of serum alanine aminotransferase (ALT) compared with those of mice receiving LdC only or normal chow (Fig. 1B). Of note, levels of aspartate aminotransferase (AST) were also significantly increased in *Nrf2^−/−^* mice receiving ethanol (Fig. S1). The AST:ALT ratio was >1, (mean AST: mean ALT was 1.63:1 for WT mice and 2.53:1 for *Nrf2^−/−^* mice) but bilirubin levels were not significantly different between the groups (data not shown). Liver sections were scored for steatosis and inflammatory activity. Representative images in Fig. 1A show that high-fat diet alone had no effect on the histological changes seen in the livers of either WT or *Nrf2^−/−^* mice over the course of 15 days. For *Nrf2^−/−^* animals fed LdC only, we observed occasional fields of view with a focus of inflammatory cells or areas of macrovesicular steatosis in animals. However, the extent of inflammation varied between individuals and, indeed, between different fields of view on individual liver sections, and was not significant when multiple fields of view were scored (Fig. 1C,D). However, the addition of ethanol caused a marked degree of steatosis, which frequently included a mixture of large and small droplets. Between alcohol-treated mice, the distribution of large droplet fat within hepatocytes was variable but steatosis overall was predominantly centrilobular in location. Semi-quantitative scoring (Fig. 1C) shows the magnitude of this response. This analysis was confirmed using Oil Red O staining (Fig. S2). As before, a mix of micro and macrovesicular steatosis was evident in mice (both WT and *Nrf2^−/−^*) that had been exposed to ethanol plus diet. This was significantly above background staining observed in livers from mice exposed to diet or chow alone. Image quantitation confirmed that steatosis was significantly increased in the presence of alcohol and diet feeding, and both WT and *Nrf2^−/−^* mice showed a similar extent of steatosis. Importantly, we did not observe hepatocyte ballooning or the presence of Mallory–Denk bodies (Denk et al., 1979; Denk et al., 2000) in any of the samples. Finally, although there was evidence of focal inflammation in some animals, total inflammation scores were not significantly different between the groups (Fig. 1D). In addition, of the groups of mice receiving ethanol, some mice occasionally demonstrated patches of necrosis upon histological assessment.

**Fig. 2.**
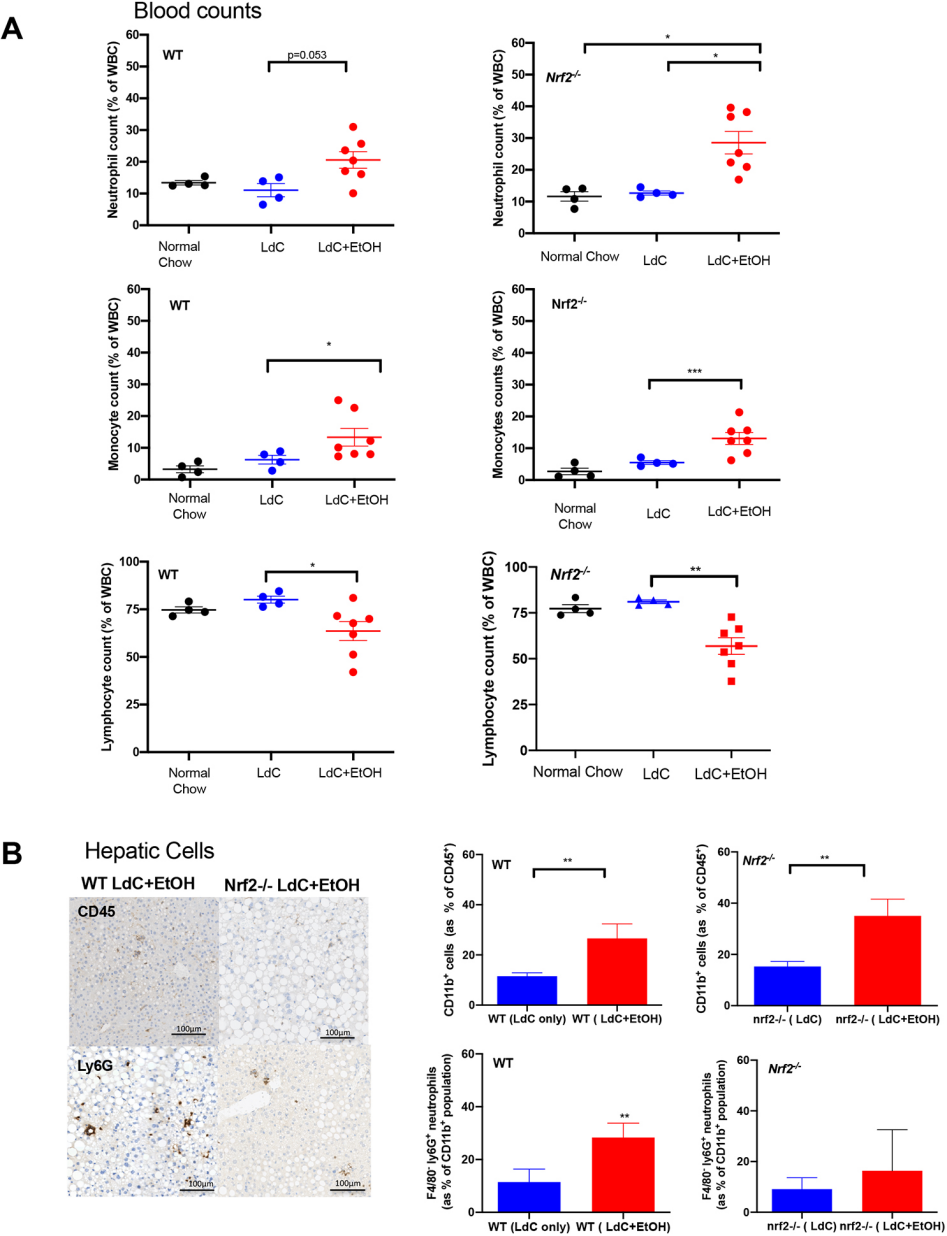
**Injured WT and *Nrf2^−/−^* mice demonstrate alterations in peripheral and hepatic immune cell populations.** (A) Following cardiac puncture EDTA anti-coagulated blood was passed through an automated blood count analyser; neutrophil, monocyte and lymphocyte counts are presented as the percentage of WBC (white blood cells). Circles represent values from individual mice and bars indicate the mean±s.e.m. of each group. Statistical differences are *P<0.05 and **P<0.01, obtained using the Kruskal–Wallis Dunn's multiple comparison test. (B) Images (left) of FFPE liver tissue sectioned (5 µm) and stained with anti-CD45, or anti-Ly6G antibodies; representatives of multiple fields of view of *n*=4-7 animals per group are shown. Graphs (right) show the cytometric analyses of digested liver lobes from mice fed an LdC or LdC+EtOH diet, collected at day 15, i.e. cytometric quantification of myeloid cells (defined by live gating and assessment of CD45^+^CD3^−^CD11b^+^ cells as a percentage of total CD45^+^ cells) and hepatic neutrophils (defined by live gating and then CD45^+^CD3^−^F4/80^−^Ly6G^+^ cells as a percentage of total CD11b^+^ cells) for WT and *Nrf2*^−/−^ mice. Bars represent the mean± s.e.m. of each group (*n*=4-7 individual livers per group. Statistical differences are *P<0.05, **P<0.01, ***P<0.001, obtained using Mann–Whitney test.

The histological sections from injured livers of our mice (Fig. 1A) suggest a trend for increased inflammatory response induced by ethanol exposure, particularly in WT animals. Analysis of full blood counts (Fig. 2A) showed a significant increase in the proportion of neutrophils and monocytes in circulating blood of both WT and *Nrf2*^−/−^ mice that were fed an LdC+EtOH diet. This was accompanied by a proportional decrease in circulating lymphocytes. Thus, we next performed a cytometric analysis of liver-resident immune cell populations, as previously described (Weston et al., 2015), and stained for the presence of these cells within liver tissue samples. In comparison to mice fed LdC alone, both WT and *Nrf2*^−/−^ mice that were fed an LdC+EtOH diet had a significantly higher proportion of CD11b-postive (i.e. CD11b^+^) cells within their liver. When using F4/80 and Ly6G antibody staining to discriminate between neutrophils and macrophages, we observed a significant increase in the recruitment of Ly6G^+^ cells to the liver after ethanol treatment, particularly in WT animals. These cells were distributed throughout the parenchyma (see Fig. 2B). We used cytometric analysis to quantify hepatic myeloid cells (defined by live gating of CD45^+^CD3^−^CD11b^+^ cells as a percentage of total CD45^+^ cells) and hepatic neutrophils (defined by live gating of CD45^+^CD3^−^F4/80^−^Ly6G^+^ cells as a percentage of total CD11b^+^ cells) for WT and *Nrf2*^−/−^ mice. We observed that the numbers of hepatic CD11b^+^ neutrophils significantly increased in both strains after exposure to LdC+EtOH. In contrast, immunohistochemical quantification of F4/80-positive macrophages/Kupffer cells and their hepatic distribution was unchanged following exposure of WT or *Nrf2*^−/−^ mice to LdC+EtOH (see Fig. 2 and quantification of staining in Fig. S3B).

**Fig. 3.**
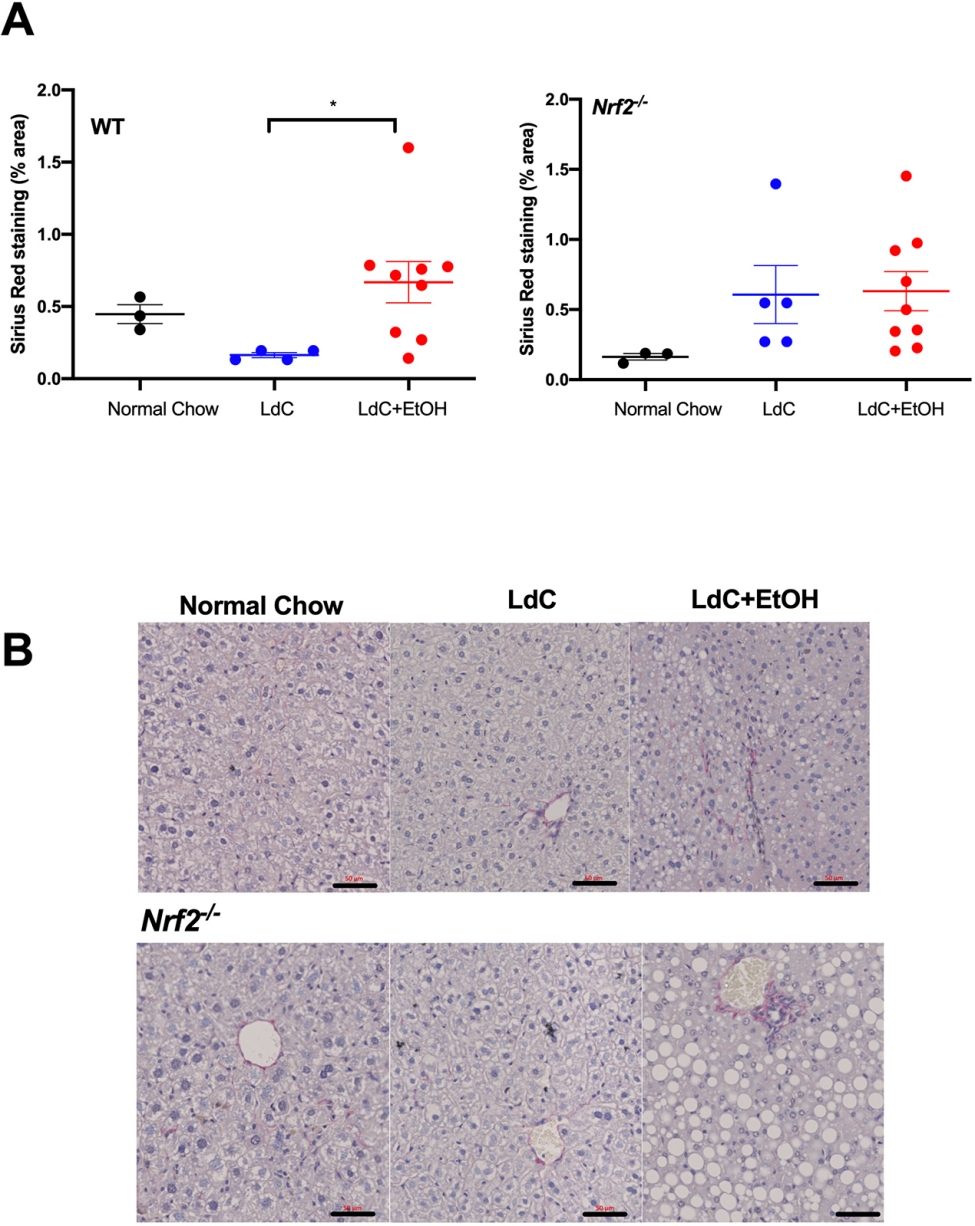
**Mice on LdC+EtOH show signs of early fibrosis at day 15.** FFPE liver tissue was sectioned (5 µm) and stained with Sirius Red. For quantification 5-6 random fields of view per section were imaged and processed with ImageJ software. Data are expressed as the percentage of the total area of the section occupied by Sirius Red staining. (A) Graphs show the average Sirius Red-stained area in indicated groups of WT and *Nrf2^−/−^* mice. Circles represent values from individual mice and bars indicate the mean±s.e.m. of each group. Statistical differences are *P<0.05, obtained using Kruskal–Wallis Dunn's multiple comparison test. (B) Representative images of Sirius Red-stained liver sections from WT and *Nrf2^−/−^* mice fed normal chow, LdC or LdC+EtOH diets. Scale bars: 50 μm; data are representative of at least *n*=3 animals per group.

Sirius Red staining of liver sections (Fig. 3) at day 15 also indicated a modest but significant accumulation of collagen in mice fed LdC+EtOH, compared to LdC alone. This was evidenced by the accumulation of stained collagenous fibrils in both the periportal areas and within the parenchyma of alcohol-exposed animals. Although the short duration of our feeding model meant that the extent of staining was modest – indicative of very early fibrogenic activation (see Fig. 3B), it is interesting that *Nrf2^−/−^* mice showed a more-variable response. Moreover, only WT mice showed a significant difference between LdC diet alone and LdC+EtOH Fig. 3A).

### LdC+EtOH induces a modest regeneration response

**Fig. 4.**
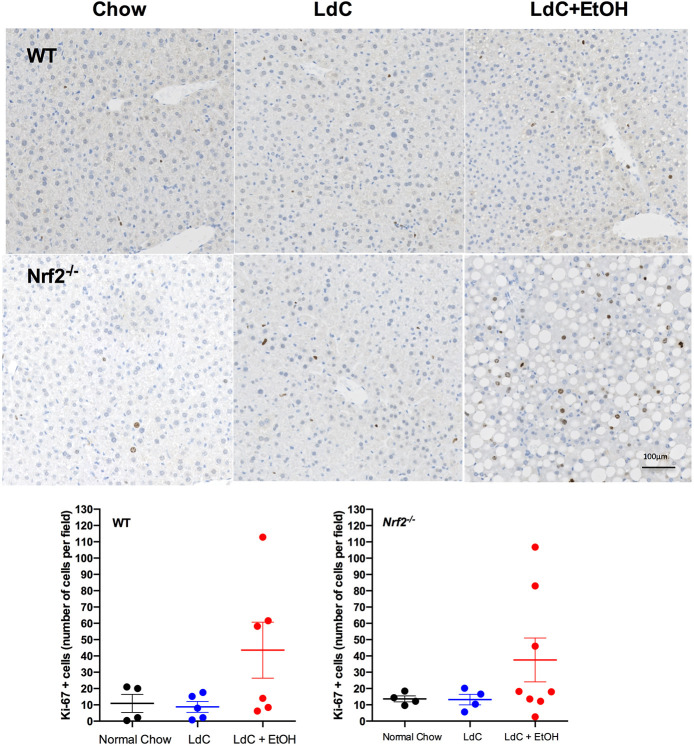
**Ethanol induces an increase in hepatocyte proliferation.** FFPE liver tissue was sectioned (5 µm) and stained with anti-Ki67 antibody to identify proliferating cells. Representative images of Ki67-stained liver sections from WT and *Nrf2^−/−^* mice fed normal chow, LdC or LdC+EtOH are shown. Scale bar: 100 μm, data are representative of at least *n*=4 animals per group. For quantification, 5-6 random fields of view per section were imaged and processed with ImageJ software. Data were expressed as number of Ki67-positive (Ki67^+^) hepatocytes per field of view.

Chronic exposure to ethanol has been shown to impair the hepatic regeneration responses (Wands et al., 1979), with decreased numbers of Ki67-positive (Ki67^+^) hepatocytes reported in patients with alcohol-related cirrhosis compared to other etiologies (Horiguchi et al., 2007). This leads to a compensatory ductular reaction in an attempt to restore the functioning hepatocyte pool (Dubuquoy et al., 2015) and, indeed, the extent of this response correlates with mortality in patients with AH (Gao and Shah, 2015; Sancho-Bru et al., 2012). To determine whether hepatocyte regeneration is triggered by our acute injury, we stained sections of matched anatomical lobes livers from WT and *Nrf2^−/−^* mice fed with chow, LdC or LdC+EtOH for Ki67 (see representative images in Fig. 4) and showed that 15 days exposure to LdC+EtOH was sufficient to stimulate hepatocyte proliferation, whereas LdC diet alone had no effect. Again, although both WT and *Nrf2^−/−^* mice exhibited a similar magnitude of response upon quantification, there was significant variation between fields of view within and between individual animals. Of note, the extent and nature of staining for Ki67 was similar at day 17 (see representative images in Fig. S4).

### Administration of LdC+EtOH alters the hepatic metabolic profile

**Fig. 5.**
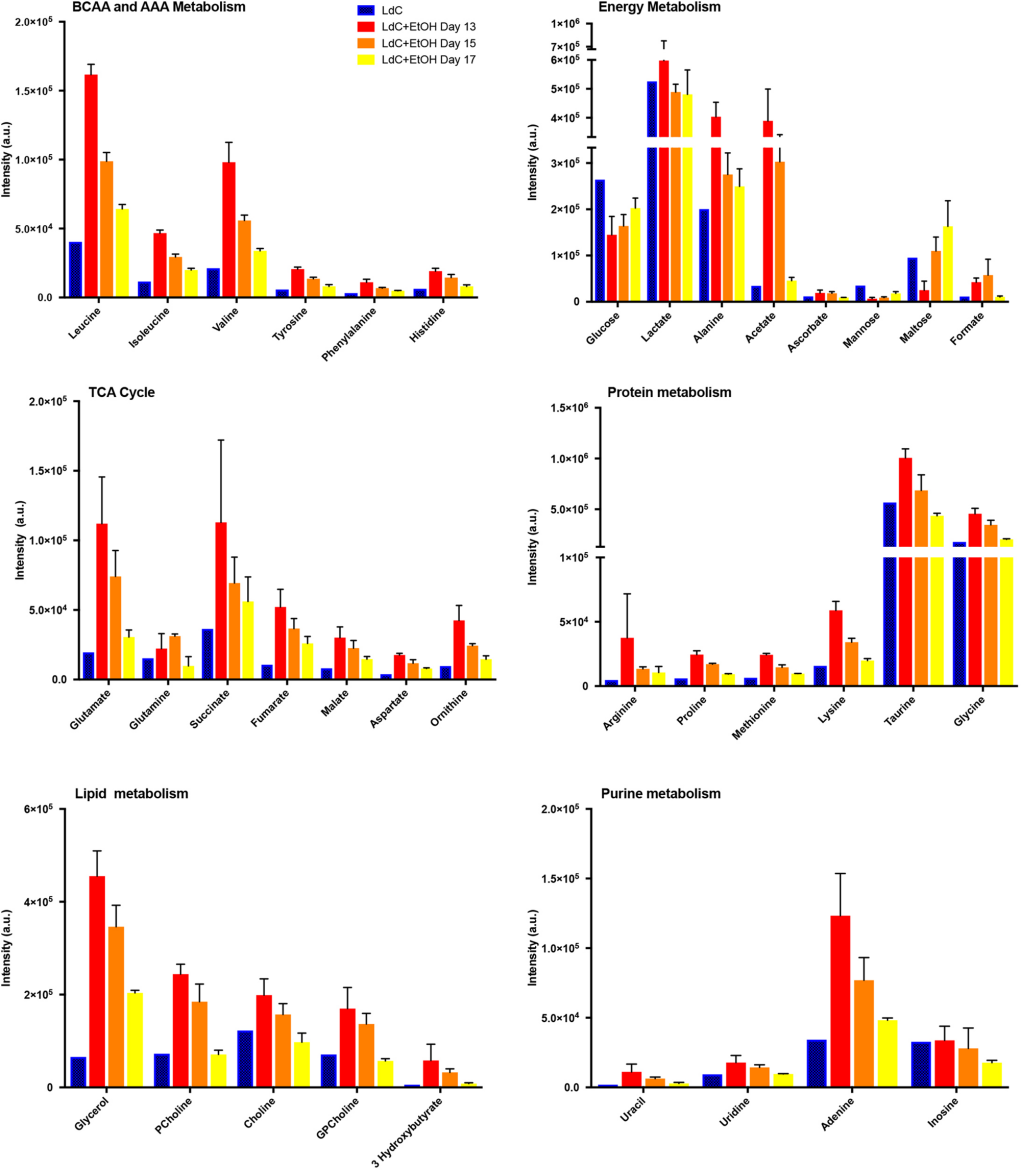
**Exposure to LdC+EtOH diet induces a transient change in hepatocyte energy metabolism.** Polar metabolites were extracted from frozen liver lobes from animals exposed to LdC diet alone at day 17 (LdC) or LdC+EtOH at days 13, 15 and 17. Data are averages of multiple samples prepared from single animals in the control groups and 3-4 animals on alcohol diets. All spectra were acquired at 300 K on a Bruker 600 MHz spectrometer with a TCI 1.7 mm z-PFG cryogenic probe using a cooled Bruker SampleJet autosampler. 1D ^1^H NMR spectra were processed using the NMRlab and Metabolab programmes within Matlab, version R2016b (MathWorks, Massachusetts, USA). Resonances were assigned using Chenomx (Alberta, Canada, 2015). Assigned peaks were then integrated in all spectra and metabolite intensities were compared between samples obtained on days as indicated, from mice exposed to LdC alone or to LdC+EtOH. Data are mean±s.e.m. of the group and have been divided into indicated categories of metabolites. Effects of diet were compared by using two-way ANOVA, which confirmed that for all classes of metabolite there was a significant effect of diet on intensity of signal (*P*<0.001 for all metabolites). Tukey's multiple comparison test was carried out to compare individual diets. Here, for all classes of metabolite control LdC was significantly different than day 13 and day 15 time points on LdC+EtOH. No significant differences were found for metabolites in LdC and LdC+EtOH at day 17. Exceptions were BCAA and AAA metabolisms, with *P*<0.001 LdC vs LdC+EtOH at day 17. AAA, aromatic amino acid; a.u., arbitrary units; BCAA, branched chain amino acid.

To characterise the mechanisms underlying the steatotic response observed in our animals we performed a detailed metabolomic characterisation using 1D-NMR on livers from WT mice fed either LdC diet alone or LdC+EtOH. Livers were collected over the final 5 days of the protocol. We analysed samples at day 13 and 15 – when the degree of injury was greatest, as well as at day 17 – when ethanol was no longer present. The LdC-diet-alone sample was collected at day 17 to allow comparison between chronic effects of diet exposure with and without ethanol. Fig. 5 shows that we were able to characterize >30 metabolites within the samples. These included amino acids, organic acids, phospholipids and nucleotides, and are divided into categories for clarity (Fig. 5). Of note, many groups of metabolites showed a clear trend for increased content in liver during peak injury, with a gradual return to baseline as ethanol was withdrawn. ANOVA confirmed a significant effect of ethanol on metabolite content. Intermediates in the metabolism of lipids, carbohydrates and amino acids were highest in ethanol-fed animals and declined to LdC-diet-alone levels by day 17. Notable exceptions included maltose and formate, the levels of which tended to increase over time, and glucose, lactate and alanine, the levels of which remained more or less static over time. Multiparametric statistical analysis confirmed that, for most metabolites, there is no significant difference between metabolite categories at day 17 in animals fed either LdC alone or LdC+ETOH. Interestingly, however, amino acid metabolism was different and, at day 17, significant differences in expression can still be seen between animals exposed to LdC-diet-alone and those that also had received ethanol (Fig. 5).

## DISCUSSION

Rodent AH models are of significant importance for new drug discovery in an area of unmet clinical need. However, many models do not recreate all systemic sequlae, fall outside of local regulatory requirements or are technically challenging to operate. Hence, each model has its own individual challenges and specific advantages (reviewed by Wilkin et al., 2016). We have based our protocol on one of the models most widely used, i.e. the NIAAA binge model (Bertola et al., 2013a), which is similarly easy to perform and involves administration of ethanol within a high-fat liquid diet and a final binge dose of ethanol before collection. This model has no mortality and no reasonable biochemical response but there is little hepatic inflammation and no fibrosis demonstrable in mice on this protocol (Bertola et al., 2013a).

This poor recreation of human disease presentation in the most widely used models has led to disappointing outcomes in clinical trials. For example, there are cases in which therapeutic anti-inflammatory targets identified as a consequence of rodent studies were unsuccessful when translated into a human setting (Woolbright and Jaeschke, 2018). Recent evidence suggested that *Nrf*2^−/−^ mice are more sensitive to oxidant-mediated injury, and exhibit a reproducible alcohol-related injury even on relatively simple dietary administration protocols. Nrf2 is a regulator of cellular responses to oxidative stress that expressed at high levels in the liver, having a protective effect in the context of alcohol exposure (Sun et al., 2018). *Nrf2*^−/−^ mice are more sensitive to the effects of ethanol, and exhibit exaggerated hypothermic and hypoglycemic responses as well as reduced capability to metabolise acetaldehyde (Sun et al., 2018). They also have a higher mortality on binge ethanol models. It is, therefore, notable that we did not see dramatic differences between injury in our WT and *Nrf2^−/−^* animals. In a previous study, Lamlé et al. (2008) used male animals and commenced the protocol with young animals (6-8 weeks). The ethanol content of the liquid diet was gradually increased – as done by us in this study – up to a maximum of 6.3% and was administered as a single daily ethanol gavage. We observed a similar weight-loss and development of steatosis but Lamlé et al. (2008) noticed that many animals became moribund after gavage and also showed significant rate of mortality (all *Nrf2^−/−^* animals died by day 24). Typical serum ALT levels exceeded 3000 IU/l in *Nrf2^−/−^* animals in the study by Lamlé. We did see this in some animals but our mean *Nrf2^−/−^* injury peaked at ∼1000 IU/l for ALT. We surmise that the difference may relate to the welfare regulations that we operate under in the UK. We cannot run experiments to a mortality endpoint and, thus, tended to start our experimental protocol with older, heavier mice (typically 10-12 weeks old, weighing ∼20 g). This led to less weight loss as a percentage of starting mass, and also meant that mice better tolerated gavages and alcohol exposure. Thus, we suggest our injury was more modest but sustainable. Similarly, our use of female animals (in both WT and *Nrf2^−/−^* groups) is likely to account for the injury in our WT cohort (in contrast to the study by Lamlé et al. (2008).

Previous studies also suggest that human females have an increased susceptibility to alcohol-related liver injury (Eagon, 2010) and that female mice tend to consume more alcohol than male mice on same protocol (Gelineau et al., 2017). Female mice have higher activities of alcohol metabolising enzymes and, thus, faster rates of ethanol metabolism and clearance compared with males (Kishimoto et al., 2002). They also have been shown to achieve higher plasma alcohol concentration, and get more-significant steatosis and triglyceride increases than males on the same protocol (Wagnerberger et al., 2013). Thus, we considered it interesting to see how female WT mice compare to *Nrf2^−^*^/−^ animals on our protocol. Our results showed that, a combination of LdC diet and ethanol combined with more-frequent additional EtOH gavage than used in the NIAAA model, reproduces many of the key features of AH in a murine model. Mice treated with ethanol developed a significant elevation in serum transaminases, with significant steatosis and a hepatic neutrophil infiltrate. This model is easier to set up than the intragastric infusion model described by Tsukamoto et al. (1985b), and is reasonably tolerated – very few mice reached the experimental or humane end-points prior to the planned completion date. Our framework for animal use in the UK means that we cannot run experiments up to a mortality endpoint. However, we did observe that the small number of animals that reached the humane endpoint is similar between WT and *Nrf2*^−/−^ animals. Interestingly, according to the parameters assessed by us, we did not see significantly greater injury in our female *Nrf2*^−/−^ mice compared to female WT mice. Nevertheless, WT females as a group showed a more-reproducible outcome within each analysis than female *Nrf2*^−/−^ mice, with virtually the same extent of injury. This might reflect the relatively mild injuries – by UK ethical standards – imposed for alcohol models but supports the concept that female mice are particularly susceptible to alcohol injury (Wagnerberger et al., 2013) and, therefore, eliminates the need to use *Nrf2*^−/−^ mice.

In our study, AST and ALT levels were more than five times above the baseline levels, with AST levels greater than those of ALT (AST>ALT). In humans, relatively modest increases in serum transaminases are seen in AH, with AST>ALT being a characteristic diagnosis criterion (Botros and Sikaris, 2013). Humans also present with elevated levels of bilirubin and hepatic neutrophil infiltration (Woolbright and Jaeschke, 2018), with fibrosis being a common feature. In agreement with many other models, we did not see elevated levels of serum bilirubin but our transaminase levels and incipient fibrosis stand apart (Woolbright and Jaeschke, 2018). In mice, neutrophils normally only constitute ∼10-25% of peripheral immune cells, so the peripheral increases in AH seen by us are significant and accompanied by a proportional decrease in circulating lymphocytes. This model induces increased hepatic recruitment of neutrophils – characteristic of ethanol-mediated liver injury – which is a prominent clinical feature of AH in humans and correlates with disease prognosis (Degré et al., 2012; Dominguez et al., 2009). Thus, we are recreating the picture seen in acute human AH, by which ethanol causes a transient increase in circulating myeloid cell populations (Gao et al., 2019), and a profound neutrophil and myeloid cell inflammation within the damaged liver. Indeed, the circulating neutrophil-to-lymphocyte ratio has prognostic relevance in AH (Forrest et al., 2019). However, we did not see particularly dense neutrophil infiltrates, abundant ballooning, cholestasis or Mallory–Denk bodies (Altamirano et al., 2014), all of which would characterise severe acute AH in humans. This is likely to relate to the short duration of our injury. It is important to note that we have quantified lymphoid and myeloid cells as a proportion of total peripheral blood or hepatic immune cells. The absolute numerical increase in hepatic cells under inflamed conditions may, therefore, be an underestimate. However, this increase was sufficient to cause systemic responses (weight loss, neutrophilia) and hepatic metabolic changes (Brandl et al., 2018; Ding et al., 2019; Latchoumycandane et al., 2014) (elevated taurine, lactate and glycerol) that do recreate the human picture.

Previous studies of mice on a high-fat diet suggest that female animals are more resistant to the metabolic sequale of these diets, i.e. with decreased weight gain, insulin secretion and better glucose tolerance than male mice (Gelineau et al., 2017). Thus, separation of effects due to an ethanol-containing, as opposed to a high-fat, diet may be easier in female animals. Our model, i.e. using female mice, incorporates administration of an LdC+EtOH diet, as it contains maltose dextrin and escalating concentrations of ethanol. This model causes a predictable steatosis driven by increased hepatic uptake of free fatty acids, *de novo* lipogenesis and impaired beta oxidation (Warner et al., 2018) as well as lipolysis, all of which is rapidly reversed on withdrawal of ethanol (Thomes et al., 2019). There appears to be a tendency for metabolites to peak at day 13 and decline thereafter, rather than continuing to increase to the final day of ethanol exposure at day 15. This might reflect different time points of sample collection within our small cohorts of animals, as there is a clear diurnal regulation of murine metabolism and feeding habits (Weger et al., 2019), which are impacted upon by ethanol exposure (Gaucher et al., 2019). The effect may also be related to time of collection after administration of final ethanol gavage. Increased energy availability in mice that were fed diet plus ethanol led to accumulation of fuel for de novo lipogenesis and the tricarboxylic acid (TCA) cycle, explaining our observed accumulation of lipid in hepatocytes. We also noticed that the response is rapidly reversible upon withdrawal of ethanol. Impaired carbohydrate metabolism and ketoacidosis are a common feature in patients admitted with AH (Wrenn et al., 1991), and features of our murine hepatic metabolic profile suggest that we are recreating this response. It has been suggested that hepatocytes preferentially use glycerol as a substrate for gluconeogenesis (Kalemba et al., 2019) and, thus, our increase in glycerol might point towards impaired glucose generation. This, combined with static lactate concentration, hints at reduced ability to generate pyruvate and inhibition of gluconeogenesis. Finally, the increased concentration of hepatic 3 hyroxybutyrate seen by us, suggests alcohol-induced ketoacidosis (Srinivasan et al., 2019) and suppression of gluconeogenesis. We observed increased accumulation of taurine during ethanol exposure, and hepatic and peripheral increases in taurine content during ethanol injury have been reported by other groups (Kerai et al., 1998). Additionally, taurine can suppress inflammation and reduce oxidative stress (Balkan et al., 2002), therefore, the hepatic increase seen by us might indicate the activation of hepatoprotective mechanisms. Consequently, our metabolic profiling supports the steatosis we observed in mice on diet and fits with the phenotype of patients exposed to alcohol. The only notable exception is the increasing hepatic abundance of maltose, which was derived from the maltose dextran used to maintain isocaloric intake in all diets.

The degree of fibrosis in our model was mild, whereas patients with AH often exhibit centrilobular pericellular/sclerosing fibrosis that may extend to cirrhosis as a consequence of ongoing acute or chronic alcohol-related injury. Unfortunately, advanced fibrosis has not been reported in any of the murine models of AH. Extending the duration or concentration of ethanol administration potentially induces a more severe fibrosis. However, these changes would be difficult to implement practically within the UK legislative framework with weight loss approaching 20% at ∼day 15. Other possible methods that could be considered to increase the severity of fibrosis include injections of carbon tetrachloride to induce fibrosis or, possibly, extending the duration of the LdC diet administration prior to giving ethanol. Given our evidence that some degree of hepatocyte regeneration occurs within our timeframe (Fig. 4), it is also possible to exacerbate the injury by compromising normal regenerative mechanisms during ongoing injury (Raven et al., 2017).

We originally hypothesized that mice deficient in *Nrf2* show an altered fibrogenic response. However, Xie et al. (2018), reported that the *Nrf2*-Keap-ARE1 pathway protects against fibrogenesis (Xie et al., 2018), Hernandez-Gea et al. (2013), showed that inhibition of *Nrf2* function in stellate cells reduces their fibrogenic potential (Hernández-Gea et al., 2013). In our hands, the degree of fibrosis assessed by PSR staining was not significantly different between WT and *Nrf2^−/−^* mice on LdC+EtOH protocol, which suggests that, at least in this model, the role is minimal. However, we did notice that the significant increase in PSR staining between LdC alone and LdC+EtOH in WT mice was not recreated in the *Nrf2^−/−^* mice (i.e. similar extent of staining appeared under both conditions), which suggest that *Nrf2* deficiency, indeed, can modify stellate cell activation. Persistent activation of *Nrf2*^−/−^ in a situation of impaired hepatic autophagy is important for inflammation, fibrogenesis and apoptosis, and is linked to tumorigenesis (Ni et al., 2014). Although deficiency in *Nrf2* may increase reactive oxygen species (ROS)-induced hepatic damage and, thus, has been linked to more-severe alcohol-dependent injury in some models (Lamlé et al., 2008), it may be unwise to use these mice when modelling injuries linked to fibrogenesis or longer term liver injury. Anecdotally, we also noticed that *Nrf2*^−/−^ mice tend to be more aggressive or at least to show diminished healing responses to injuries sustained in territorial fights. This is in keeping with reports of impaired wound healing responses in this strain (Li et al., 2019; Long et al., 2016) and influenced our opinions on the relative merits of using this strain for modelling alcohol injury. However, we note that the responses observed were generated from relatively small cohorts of animals housed in our specific facility and, thus, it is possible that the use of increased numbers of animals or housing of animals in a different facility to ours could explain differential sensitivities of the *Nrf2* strain in different models.

There are a number of challenges when modelling human AH in mice. The alcohol catabolism rate is up to five-times higher in rodents compared to humans, and the amount of ethanol administered to animals in order to achieve sustained blood alcohol concentration and subsequent liver injury cannot be directly compared with human alcohol consumption (Holmes et al., 1986). In a time-course experiment, we found that, upon withdrawal of ethanol at day 15 of this protocol, mice had normalised serum transaminase levels and were gaining weight again by day 17. Similarly, our reported metabolic changes began to normalise by day 17. Such rapid resolution of liver injury would atypical of patients with severe AH. However, our use of female WT mice has recreated many of the key features of human AH and, thus, has potential to improving our understanding of the pro-inflammatory, profibrotic and metabolic disturbances that characterise this human disease.

## MATERIALS AND METHODS

### LdC diet and EtOH feeding

All mice were maintained and housed under conventional conditions in the Biomedical Services Unit at the University of Birmingham, UK. All animal experiments were performed under a Home Office project license in accordance with UK legislation, and studies were approved by the local ethical review board. Specific pathogen-free female 8- to 10-week-old WT and *Nrf2^−/−^* mice on a *C57/BL6J* background*,* were obtained from the Charles Rivers Laboratories (Margate, UK; https://guide.labanimal.com/supplier/charles-river-uk-ltd). Female WT and *Nrf2^−/−^* mice were fed LdC liquid diet (Special Diet Services, UK) *ad libitum* for 5 days via specialised feeding tubes (Richter tubes). Preparation of the diet involved using a hand-held blender to mix a set volume of dry mix with maltose dextrin and H_2_O (see Table 1).

**Table 1.**
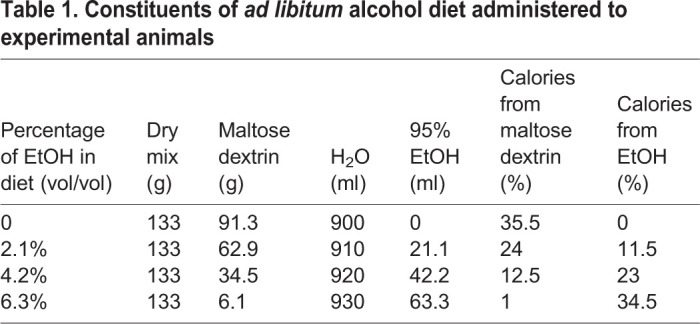
**Constituents of *ad libitum* alcohol diet administered to experimental animals**

For experimental groups LdC diet was supplemented with escalating doses of ethanol (EtOH, 95%, William Hodgson and Company, UK) over the following week (2.1% EtOH on day 7 increased to 4.2% at day 9, and 6.2% at day 12). All experimental mice were additionally gavaged twice daily with 33% of EtOH diluted with H_2_O from day 6 until day 15. Prior to gavage we completed a mouse physiological condition assessment sheet and the volume administered to healthy animals was determined by weight (190 μl for mice 19 g, 200 µl for mice 20 g, 250 µl for mice >25 g). Control mice were fed LdC diet without EtOH throughout the experiment with the amount of maltodextrin adjusted to maintain total calorie load in both groups (see Table 1). Blood and liver tissue were collected at the end of the experiment. Alanine aminotransferase (ALT), aspartate aminotransferase (AST), alkaline phosphatase (ALP) and bilirubin levels in murine serum as well as full blood count and differential were determined utilising clinical grade automated analysers at Birmingham Women's Hospital NHS Foundation Trust, Birmingham, UK.

### Flow cytometry

Liver infiltrating immune cells were isolated from freshly harvested liver lobes. The liver was flushed with PBS before isolation to remove blood cells. The tissue was weighed and manually digested using a 70 μm strainer (Falcon). The filtrate was diluted in cold RPMI and centrifuged twice at 700 ***g*** for 5 min. A working dilution of Optiprep (Sigma) at 1.09 g/ml was obtained by mixing neat Optiprep with PBS at a 4:11 ratio. 5 ml of cell suspension was layered carefully over 7 ml of Optiprep solution, and centrifuged at 1000 ***g*** for 25 min. The mononuclear cells at the interface were washed twice and resuspended in PBS+1% fetal calf serum. Cells were stained for 30 min in the dark with zombie (APC-Cy7 in a 1:1000 concentration) to assess live/dead status of cells. Samples were washed and incubated for 20 min with purified anti-mouse CD16/32 antibody (E-bioscience, clone 93, 1:25 dilution of manufacturers stock) to block non-specific Fc- receptor binding. Finally, cells were incubated for 30 min with lineage specific antibodies for identification of myeloid and lymphoid cells (see Table 2).

**Table 2.**
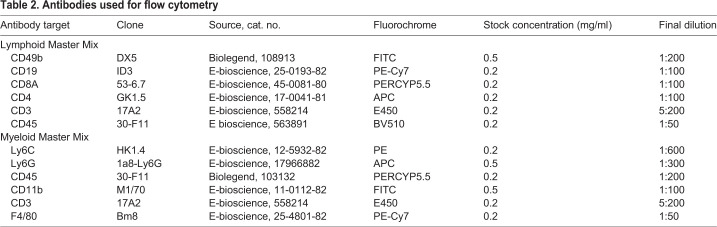
**Antibodies used for flow cytometry**

### Immunohistochemistry and histological analysis

Haematoxylin and eosin (H&E) staining was performed on formalin-fixed, paraffin-embedded sections from each mouse liver using standard protocols. Sections were reviewed by an experienced liver pathologist (S.G.H.), blinded to the experimental conditions. For each case the pattern (large or small droplet), zonal distribution and severity of steatosis were recorded. Disease severity was scored semi-quantitatively for the overall degree of steatosis (0-3), the amount of large droplet steatosis (0-3), and the severity of inflammation according to the Kleiner system (Kleiner et al., 2005). For antibody staining, formalin-fixed paraffin-embedded (FFPE) sections were deparaffinised with xylene and then rehydrated through graded alcohol prior to high-temperature antigen retrieval in pre-heated EDTA buffer (pH 8) for 15 min. Endogenous peroxidase activity was blocked (Dako-Peroxidase, Dako Ltd, Cambridge UK) for 10 min, and non-specific binding of the antibodies to the sections was prevented by incubation with ×10 casein (Vector Labs, Burlingame, CA, USA) diluted in PBS. Sections were incubated in primary antibody, or an isotype matched control for 1 h at room temperature in a humidified chamber on a rocker. They were then washed twice, followed by addition of the relevant horse-radish peroxidase conjugated secondary antibody for 30 min and signal development using ImmPACT DAB reagent (Vector Labs). Sections were washed, counterstained with Mayer's haematoxylin, dehydrated with 99% alcohol, cleared with xylene and then mounted in DPX (Leica GmbH). The exact conditions were optimised for each antibody (see Table 3).

**Table 3.**
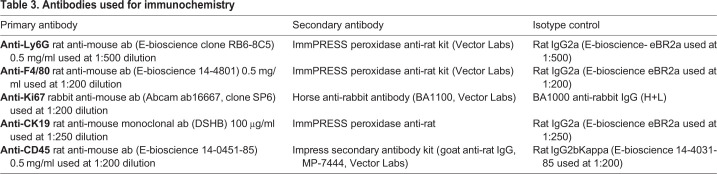
**Antibodies used for immunochemistry**

For image analysis, at least five fields of view were taken per sample using bright-field microscopy with AxioScanner software at ×20 magnification. The images were reviewed on ImageJ software version 1.42 (NIH). The number of Ki67^+^ hepatocytes per field was counted, as were the number of cells staining positively for Ly6G (Ly6G^+^ cells). For CD45 and F4/80 antibody staining (Table 3), morphometric analysis was performed.

### Sirius Red and Oil Red O staining

Deparaffinized, hydrated sections were placed into 5% phosphomolybdic acid solution (diluted in distilled H_2_O) for 5 min. Sections were washed with PBS and submerged in 0.1% Sirius Red (Direct Red 80 dissolved in saturated picric acid solution) for 90 min on a rocker. The slides were dipped in acidified H_2_O (0.5% glacial acetic acid) for 30 s twice, followed by 100% EtOH 3× for 30 s each. Finally staining intensity was determined using morphometric analysis via ImageJ software version 1.42 (NIH), using 5 non-overlapping fields selected at random from each mouse (at ×20 magnification). To quantify lipid content in hepatocytes, fresh frozen tissue sections were incubated in 60% isopropanol for 5 min followed by Oil Red O reagent for 15 min at room temperature. This was tipped off and 60% isopropanol was added for another 5 min. Slides were washed twice with H_2_O and finally mounted using aqueous mountant (Thermoscientific, Shandon). Sections were imaged using bright-field microscopy and % staining area was determined using morphometric analysis via ImageJ software version 1.42 (NIH), using 5 non-overlapping fields selected at random from each mouse (at ×20 magnification).

### Assessment of liver metabolism by NMR

Single lobes from mouse livers were collected at day 13, 15 and 17 for mice exposed to LdC+EtOH feeding. Matched livers from mice fed control LdC diet alone were collected on day 17. Tissue was immediately snap-frozen upon collection and stored at −80°C until processing. To prepare extracts for NMR analysis, ∼100 mg liver tissue was added to gentleMACs M-Tube in cold methanol (8 µl/mg) and purified H_2_O (2 µl/mg). Samples were homogenised using a gentleMACs homogenizer (Miltenyi, UK) and polar metabolites were extracted as described previously (Schofield et al., 2017) Samples were kept at 4°C prior to NMR. All spectra were acquired at 300 K on a Bruker 600 MHz spectrometer with a TCI 1.7 mm z-PFG cryogenic probe using a cooled Bruker SampleJet autosampler as previously described (Saborano et al., 2019).1D ^1^H NMR spectra were processed using the NMRlab and Metabolab programmes within MATLAB (MathWorks, Massachusetts, USA). Following Fourier transformation, spectra were phased, referenced to TMSP δ 0.00 ppm, baseline corrected and the water region was excluded together with the edges of the spectrum void of signal. Lastly, spectra were scaled to the total spectral area and resonances were assigned using Chenomx (Alberta, Canada, 2015), and by consulting the NMR metabolic profiling human metabolome database (HMDB). Further details can be found in Saborano et al. (2019).

## Supplementary Material

Supplementary information
