# Supplementary material for: Alcoholic hepatitis and metabolic disturbance in female mice: a more tractable model than Nrf2−/− animals
Source: Dis Model Mech. 2020 Dec 29;13(12):dmm046383. doi: 10.1242/dmm.046383 (PMC7790192; doi:10.1242/dmm.046383)
Supplement: Supplementary information [file dmm-13-046383-s1.pdf]

Supplemental Figure 1

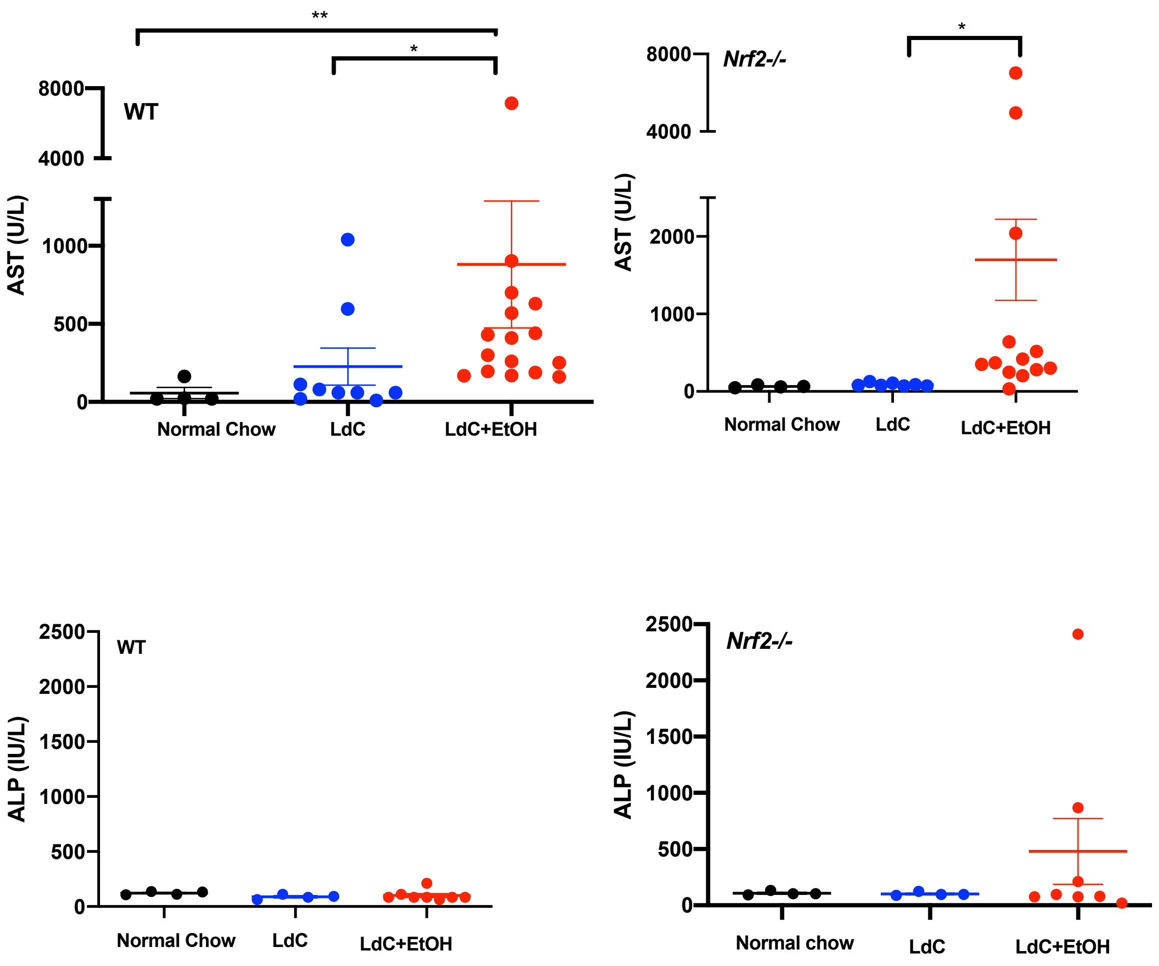

Figure S1 : Serum biochemical analysis from mice treated with modified LdC diet and ethanol WT and *Nrf2*$^{-/-}$ mice were fed LdC diet only for 5 days. At day 6 experimental groups received 2.2% EtOH mixed into the LdC diet. This was increased to 4.4% at day 9, and to 6.3% at day 12. Additionally, experimental groups received twice daily ethanol gavage of 33% EtOH (LdC+EtOH). Control mice received LdC diet only throughout the experiment and were not gavaged. Serum was prepared from WT and *Nrf2*$^{-/-}$ mice at day 15 for determination of ALST and AP concentration. Symbols are values from individual mice and bars are mean ±SEM for the group. Statistical difference level is indicated by * for $p<0.05$ and ** for $p<0.01$ performed with the Kruskal-Wallis Dunn's multiple comparison test.

Supplemental Figure 2

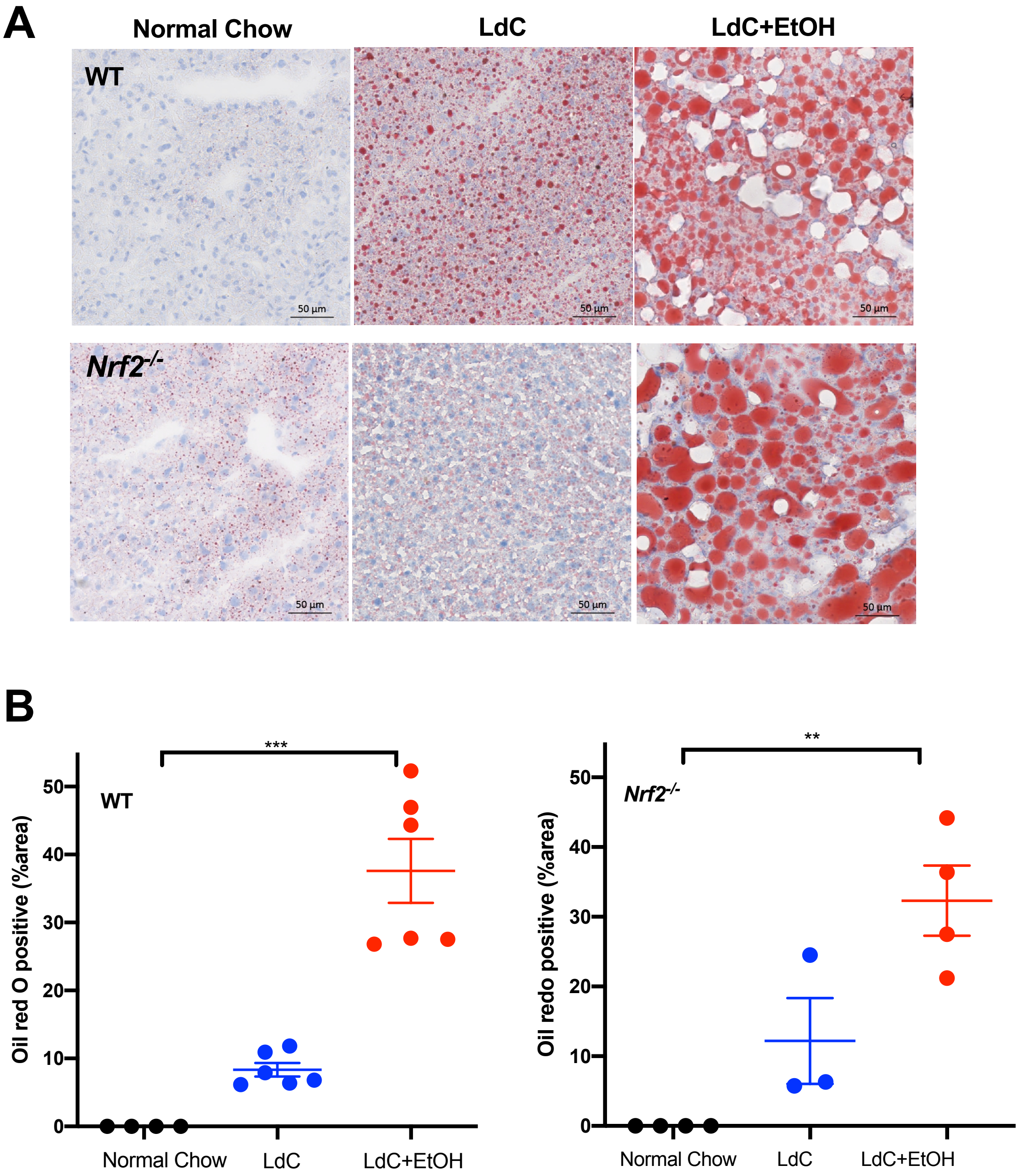

Figure S2: Mice fed ethanol-containing diet demonstrate significant liver steatosis. Accumulation of lipid droplets in hepatocytes within murine liver tissue sections was assessed by Oil red O staining. (A) Representative Oil Red O-stained liver sections from WT and *Nrf2*$^{-/-}$ mice fed normal chow, LdC or LdC+EtOH are shown. Scale bar is 50µm, data is representative of at least n=4 animals per group. (B) Quantification of extent of Oil Red O staining. Data form multiple fields of view per animal were captured using ImageJ and are expressed as a percentage of surface area occupied by lipid. Symbols are values from individual mice and bars represent mean ±SEM of the group. Statistical difference level is indicated by ** for p<0.01 or ***p<0.001 based on Kruskal-Wallis Dunn's multiple comparison test.

Supplemental Figure 3

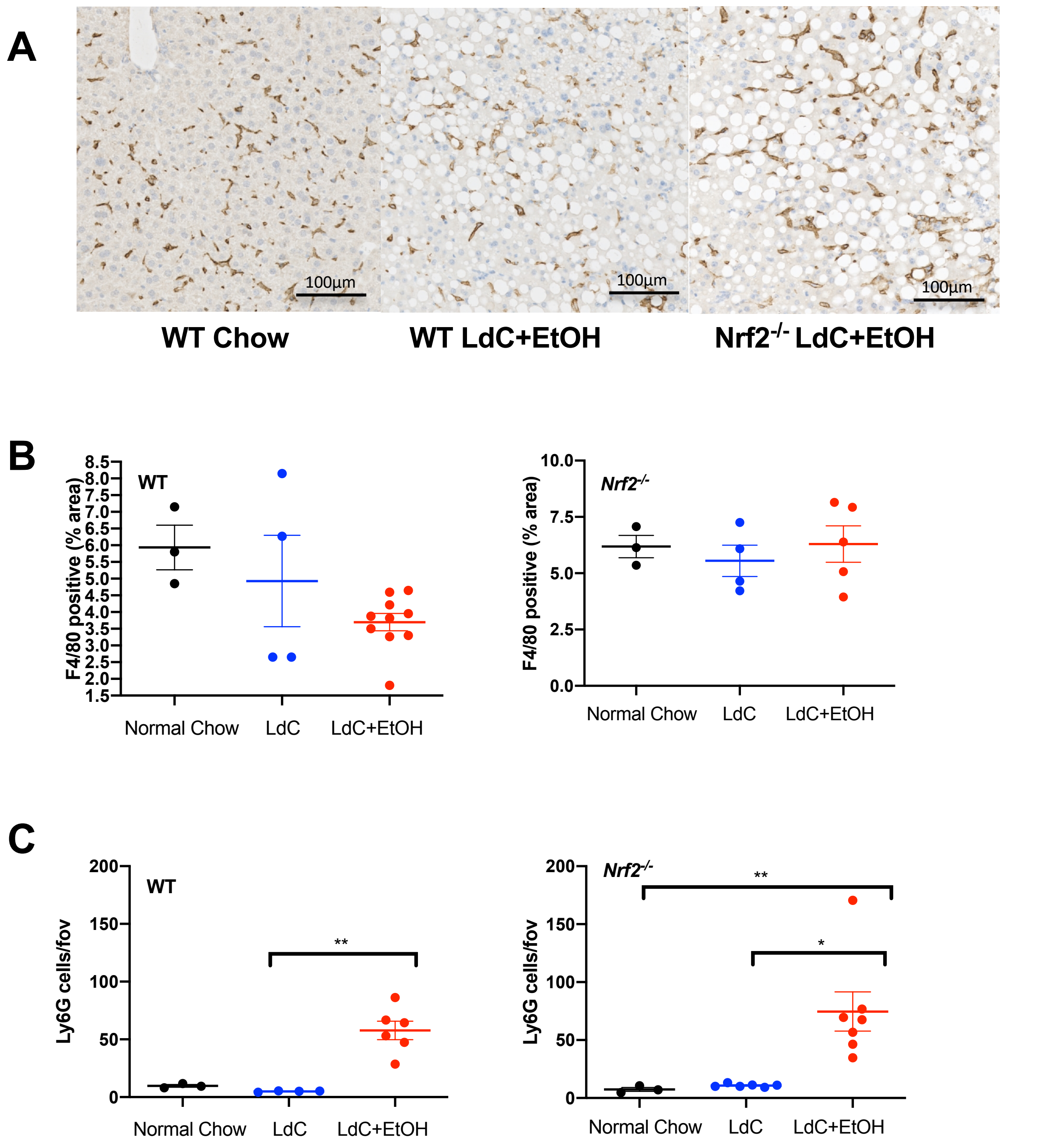

Figure S3: Staining for F4/80 positive cells suggests no change in number after exposure to ethanol. A : Representative images of F4/80+ cells in liver sections from WT and

Nrf2$^{-/-}$ mice fed chow or LdC+EtOH as indicated, representative of at least n=3 animals per group. (B and C) Quantification of extent of F480 or Ly6G staining. Data from multiple fields of view per animal were captured using ImageJ and are expressed as a percentage of surface area occupied by F4/80 staining or number of Ly6G positive cells per field.Symbols are mean values from individual mice and bars represent mean ±SD of the group. Statistical difference level is indicated by ** for p<0.01 based on Kruskal-Wallis Dunn’s multiple comparison test.

Supplemental Figure 4: Staining for Ki67 positive cells 2 days after withdrawal of ethanol ethanol. Representative images from three individual animals where livers were harvested at day 17. Scale bar represents 100um and brown stained Ki67+ cells are distributed across the parenchyma in all cases.

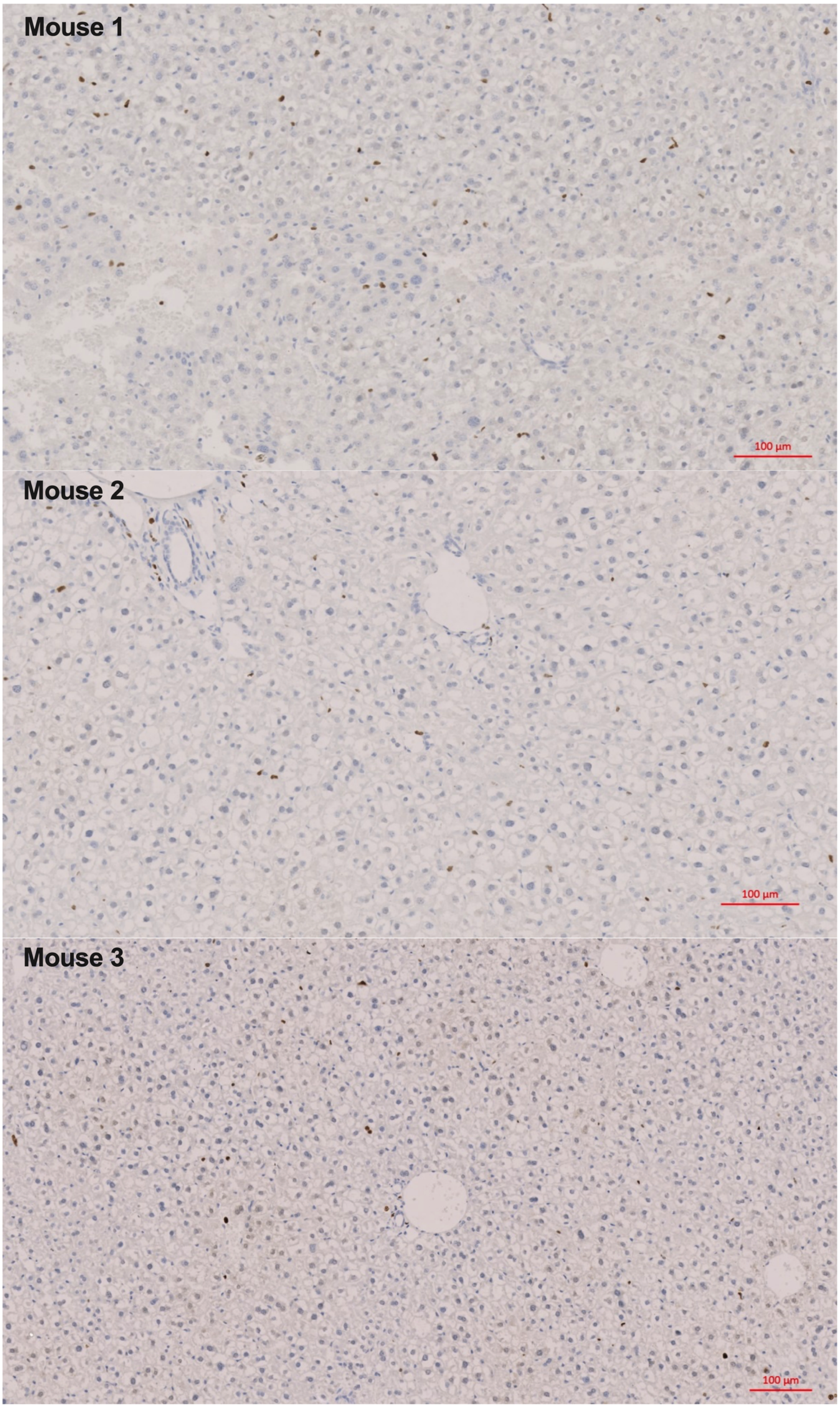

Disease Models & Mechanisms • Supplementary information
